# Bis[4-(2-hy­droxy­benzyl­idene­amino)­benzoato-κ*O* ^1^]tetra­kis­(methanol-κ*O*)cadmium

**DOI:** 10.1107/S1600536811015364

**Published:** 2011-05-07

**Authors:** Ying Wang, Xia Wang, Yu-Xian Li, Huai-Xia Yang, Yan-Ju Liu

**Affiliations:** a Department of Geriatrics, The First Affiliated Hospital, Zhengzhou University, Zhengzhou 450000, People’s Republic of China; b Pharmacy College, Henan University of Traditional Chinese Medicine, Zhengzhou 450008, People’s Republic of China

## Abstract

In the title mononuclear complex, [Cd(C_14_H_10_NO_3_)_2_(CH_3_OH)_4_], the Cd^2+^ cation is situated on an inversion centre. It exhibits a distorted octa­hedral coordination, defined by two carboxyl­ate O atoms from two monodentate anions and by four O atoms from four methanol mol­ecules. The crystal structure comprises intra­molecular O—H⋯O and O—H⋯N, and inter­molecular O—H⋯O hydrogen bonds. The latter help to construct a layered structure extending parallel to (100).

## Related literature

For background to Schiff base ligands, see: Garnovskii *et al.* (1993 ▶); Banerjee *et al.* (2004 ▶); Zhong *et al.* (2009 ▶). For background to cadmium complexes, see: Meng *et al.* (2004 ▶); Wang *et al.* (2010 ▶).
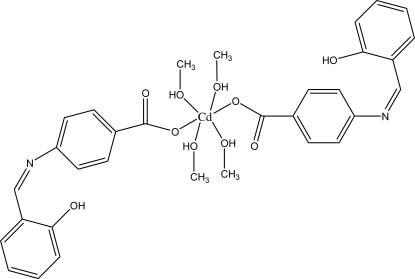


## Experimental

### 

#### Crystal data

[Cd(C_14_H_10_NO_3_)_2_(CH_4_O)_4_]
*M* *_r_* = 721.04
Monoclinic, 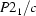

*a* = 15.564 (3) Å
*b* = 11.937 (2) Å
*c* = 8.8946 (18) Å
β = 99.69 (3)°
*V* = 1629.0 (6) Å^3^
*Z* = 2
Mo *K*α radiation
μ = 0.73 mm^−1^
*T* = 293 K
0.21 × 0.19 × 0.16 mm

#### Data collection

Rigaku Saturn diffractometer
Absorption correction: multi-scan (*CrystalClear*; Rigaku/MSC, 2006 ▶) *T*_min_ = 0.862, *T*_max_ = 0.892
7793 measured reflections
2760 independent reflections
2137 reflections with *I* > 2σ(*I*)
*R*_int_ = 0.055

#### Refinement

*R*[*F* ^2^ > 2σ(*F* ^2^)] = 0.072
*wR*(*F* ^2^) = 0.166
*S* = 1.13
2760 reflections
202 parameters
H-atom parameters constrained
Δρ_max_ = 0.97 e Å^−3^
Δρ_min_ = −0.63 e Å^−3^

### 

Data collection: *CrystalClear* (Rigaku/MSC, 2006 ▶); cell refinement: *CrystalClear*; data reduction: *CrystalClear*; program(s) used to solve structure: *SHELXS97* (Sheldrick, 2008 ▶); program(s) used to refine structure: *SHELXL97* (Sheldrick, 2008 ▶); molecular graphics: *XP* in *SHELXTL* (Sheldrick, 2008 ▶); software used to prepare material for publication: *SHELXTL*.

## Supplementary Material

Crystal structure: contains datablocks global, I. DOI: 10.1107/S1600536811015364/wm2478sup1.cif

Structure factors: contains datablocks I. DOI: 10.1107/S1600536811015364/wm2478Isup2.hkl

Supplementary material file. DOI: 10.1107/S1600536811015364/wm2478Isup3.cdx

Additional supplementary materials:  crystallographic information; 3D view; checkCIF report

## Figures and Tables

**Table 1:** Selected bond lengths (Å)

Cd1—O1	2.230 (5)
Cd1—O4	2.295 (5)
Cd1—O3	2.315 (5)

**Table 2:** Hydrogen-bond geometry (Å, °)

*D*—H⋯*A*	*D*—H	H⋯*A*	*D*⋯*A*	*D*—H⋯*A*
O4—H4⋯O2	0.87	1.87	2.653 (7)	150
O5—H5⋯N1	0.82	1.90	2.632 (9)	148
O3—H3⋯O2^i^	0.85	1.83	2.640 (7)	160

Symmetry code: (i) 
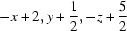
.

**Fig. 1.**
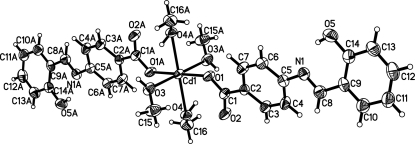
View of the molecular structure of (I), showing the labelling of the non-H atoms and atomic displacement parameters at the 30% probability level. [Symmetry code: A) -x+2, -y+1, -z+3.]

**Fig. 2.**
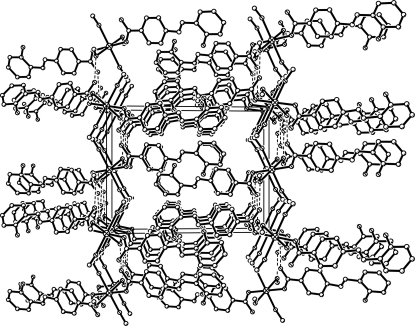
View of the packing of the structure of (I), showing intermolecular hydrogen bonding (dotted lines). H atoms have been omited for clarity.

## supplementary crystallographic information

### Comment

Schiff base ligands played an important role in the development of coordination chemistry due to their metal binding ability (Garnovskii *et al.*, 1993). Schiff bases and their metal complexes have numerous applications in biological systems and material sciences (Banerjee *et al.*, 2004; Zhong *et al.*, 2009). The Cd^2+^ ion is a good model atom to construct complexes owing to its property to form bonds with different types of donors simultaneously, and to its various coordination modes (Meng *et al.*, 2004; Wang *et al.*, 2010). In this work, we describe the synthesis and structure of the title complex, [Cd(C_14_H_10_NO_3_)_2_(CH_3_OH)_4_], (I).

In complex (I), the Cd^2+^ ion is situated on an inversion centre and is six-coordinated by two carboxylate O atoms from two monodentate ligands and by four O atoms from four methanol molecules (Fig. 1). The dihedral angle between the phenyl ring and the benzylidenimino moiety is 23.4 (4) °.

The crystal structure of (I) comprises intramolecular O—H···O and O—H···N hydrogen bonds that help to stabilize the molecular conformation. Intermolecular O—H···O hydrogen bonds between methanol molecules and the free carboxylate O atoms of neighbouring molecules construct a layered structure extending parallel to (100), as shown in Fig. 2.

### Experimental

*N*-(4-carboxyphenyl)salicylideneimine (0.04 mmol, 0.0097 g) in methanol (6 ml) was added dropwise to a methanol solution (5 ml) of CdCl_2_ (0.02 mmol, 0.0037 g) in methanol. The resulting solution was allowed to stand at room temperature. After two weeks good quality crystals with pale yellow colour were obtained and were dried in air.

### Refinement

H atoms bound to C atoms were generated geometrically and refined as riding atoms with C—H = 0.93Å and U_iso_(H) = 1.2×U_eq_(C). H atoms bound to O atoms were found from difference maps and refined with distance restraints between 0.82—0.87 Å and U_iso_(H) = 1.5×U_eq_(O).

### Crystal data

**Table:**

[Cd(C_14_H_10_NO_3_)_2_(CH_4_O)_4_]	*F*(000) = 740
*M**_r_* = 721.04	*D*_x_ = 1.470 Mg m^−^^3^
Monoclinic, *P*2_1_/*c*	Mo *K*α radiation, λ = 0.71073 Å
Hall symbol: -P 2ybc	Cell parameters from 3109 reflections
*a* = 15.564 (3) Å	θ = 2.2–28.0°
*b* = 11.937 (2) Å	µ = 0.73 mm^−^^1^
*c* = 8.8946 (18) Å	*T* = 293 K
β = 99.69 (3)°	Prism, pale yellow
*V* = 1629.0 (6) Å^3^	0.21 × 0.19 × 0.16 mm
*Z* = 2	

### Data collection

**Table:**

Rigaku Saturn diffractometer	2760 independent reflections
Radiation source: fine-focus sealed tube	2137 reflections with *I* > 2σ(*I*)
graphite	*R*_int_ = 0.055
Detector resolution: 28.5714 pixels mm^-1^	θ_max_ = 25.0°, θ_min_ = 2.2°
ω scans	*h* = −18→18
Absorption correction: multi-scan (*CrystalClear*; Rigaku/MSC, 2006)	*k* = −14→10
*T*_min_ = 0.862, *T*_max_ = 0.892	*l* = −10→9
7793 measured reflections	

### Refinement

**Table:**

Refinement on *F*^2^	Primary atom site location: structure-invariant direct methods
Least-squares matrix: full	Secondary atom site location: difference Fourier map
*R*[*F*^2^ > 2σ(*F*^2^)] = 0.072	Hydrogen site location: inferred from neighbouring sites
*wR*(*F*^2^) = 0.166	H-atom parameters constrained
*S* = 1.13	*w* = 1/[σ^2^(*F*_o_^2^) + (0.0483*P*)^2^ + 5.4285*P*] where *P* = (*F*_o_^2^ + 2*F*_c_^2^)/3
2760 reflections	(Δ/σ)_max_ < 0.001
202 parameters	Δρ_max_ = 0.97 e Å^−^^3^
0 restraints	Δρ_min_ = −0.63 e Å^−^^3^

### Special details

**Table:**

Geometry. All e.s.d.'s (except the e.s.d. in the dihedral angle between two l.s. planes) are estimated using the full covariance matrix. The cell e.s.d.'s are taken into account individually in the estimation of e.s.d.'s in distances, angles and torsion angles; correlations between e.s.d.'s in cell parameters are only used when they are defined by crystal symmetry. An approximate (isotropic) treatment of cell e.s.d.'s is used for estimating e.s.d.'s involving l.s. planes.
Refinement. Refinement of *F*^2^ against ALL reflections. The weighted *R*-factor *wR* and goodness of fit *S* are based on *F*^2^, conventional *R*-factors *R* are based on *F*, with *F* set to zero for negative *F*^2^. The threshold expression of *F*^2^ > σ(*F*^2^) is used only for calculating *R*-factors(gt) *etc*. and is not relevant to the choice of reflections for refinement. *R*-factors based on *F*^2^ are statistically about twice as large as those based on *F*, and *R*- factors based on ALL data will be even larger.

### Fractional atomic coordinates and isotropic or equivalent isotropic displacement parameters (Å^2^)

**Table:**

	*x*	*y*	*z*	*U*_iso_*/*U*_eq_	
Cd1	1.0000	0.5000	1.5000	0.0478 (3)	
N1	0.5587 (4)	0.4273 (6)	0.7851 (7)	0.0592 (17)	
O1	0.8888 (4)	0.4749 (4)	1.3094 (6)	0.0591 (15)	
O2	0.9173 (4)	0.3047 (4)	1.2333 (7)	0.0740 (18)	
O3	1.0768 (4)	0.5897 (4)	1.3338 (6)	0.0684 (16)	
H3	1.0698	0.6603	1.3269	0.103*	
O4	1.0614 (3)	0.3361 (4)	1.4350 (6)	0.0605 (14)	
H4	1.0252	0.3053	1.3620	0.091*	
O5	0.4108 (4)	0.5348 (5)	0.7011 (8)	0.0810 (19)	
H5	0.4577	0.5198	0.7542	0.122*	
C1	0.8710 (5)	0.3913 (6)	1.2257 (8)	0.0513 (18)	
C2	0.7912 (5)	0.3969 (6)	1.1089 (7)	0.0467 (17)	
C3	0.7585 (5)	0.3037 (6)	1.0221 (9)	0.062 (2)	
H3A	0.7881	0.2358	1.0373	0.075*	
C4	0.6827 (5)	0.3101 (6)	0.9135 (9)	0.066 (2)	
H4A	0.6622	0.2472	0.8570	0.079*	
C5	0.6378 (5)	0.4124 (6)	0.8902 (8)	0.0519 (19)	
C6	0.6693 (5)	0.5042 (7)	0.9765 (9)	0.061 (2)	
H6	0.6398	0.5722	0.9619	0.073*	
C7	0.7443 (6)	0.4964 (6)	1.0843 (8)	0.062 (2)	
H7	0.7638	0.5592	1.1419	0.074*	
C8	0.5382 (5)	0.3639 (7)	0.6702 (9)	0.063 (2)	
H8	0.5776	0.3100	0.6491	0.076*	
C9	0.4532 (5)	0.3738 (7)	0.5696 (9)	0.059 (2)	
C10	0.4316 (6)	0.2979 (8)	0.4527 (10)	0.076 (3)	
H10	0.4725	0.2449	0.4347	0.092*	
C11	0.3504 (7)	0.2990 (8)	0.3616 (11)	0.084 (3)	
H11	0.3353	0.2445	0.2870	0.101*	
C12	0.2932 (6)	0.3808 (9)	0.3826 (11)	0.082 (3)	
H12	0.2400	0.3841	0.3170	0.099*	
C13	0.3112 (6)	0.4603 (8)	0.4994 (11)	0.076 (3)	
H13	0.2700	0.5136	0.5149	0.091*	
C14	0.3917 (6)	0.4568 (7)	0.5904 (9)	0.062 (2)	
C15	1.0839 (5)	0.5457 (5)	1.1902 (7)	0.091 (3)	
H15A	1.1181	0.5955	1.1392	0.137*	
H15B	1.0269	0.5378	1.1305	0.137*	
H15C	1.1118	0.4738	1.2027	0.137*	
C16	1.1049 (5)	0.2536 (5)	1.5305 (7)	0.079 (3)	
H16A	1.1236	0.1942	1.4708	0.118*	
H16B	1.0663	0.2241	1.5943	0.118*	
H16C	1.1547	0.2867	1.5931	0.118*	

### Atomic displacement parameters (Å^2^)

**Table:**

	*U*^11^	*U*^22^	*U*^33^	*U*^12^	*U*^13^	*U*^23^
Cd1	0.0584 (5)	0.0351 (4)	0.0469 (4)	0.0027 (4)	0.0006 (3)	−0.0028 (3)
N1	0.055 (4)	0.069 (4)	0.053 (4)	0.000 (3)	0.004 (3)	0.003 (3)
O1	0.064 (4)	0.047 (3)	0.061 (3)	0.006 (2)	−0.005 (3)	−0.015 (2)
O2	0.073 (4)	0.047 (3)	0.090 (4)	0.010 (3)	−0.022 (3)	−0.015 (3)
O3	0.098 (5)	0.048 (3)	0.063 (3)	−0.011 (3)	0.023 (3)	0.006 (2)
O4	0.063 (4)	0.043 (3)	0.069 (3)	0.011 (2)	−0.006 (3)	−0.008 (2)
O5	0.064 (4)	0.086 (4)	0.094 (5)	0.018 (3)	0.015 (4)	−0.003 (3)
C1	0.060 (5)	0.047 (4)	0.046 (4)	−0.004 (4)	0.005 (4)	0.000 (3)
C2	0.045 (4)	0.054 (4)	0.041 (4)	−0.008 (3)	0.006 (3)	−0.001 (3)
C3	0.066 (6)	0.046 (4)	0.069 (5)	0.001 (4)	−0.004 (4)	−0.003 (4)
C4	0.066 (6)	0.053 (4)	0.068 (5)	−0.007 (4)	−0.018 (4)	−0.006 (4)
C5	0.051 (5)	0.060 (5)	0.045 (4)	−0.001 (4)	0.006 (4)	0.008 (3)
C6	0.064 (5)	0.059 (4)	0.056 (5)	0.006 (4)	−0.001 (4)	0.000 (4)
C7	0.085 (6)	0.050 (4)	0.045 (4)	0.000 (4)	−0.007 (4)	0.001 (3)
C8	0.057 (5)	0.067 (5)	0.065 (5)	0.004 (4)	0.008 (4)	0.003 (4)
C9	0.043 (5)	0.070 (5)	0.060 (5)	−0.007 (4)	0.001 (4)	0.013 (4)
C10	0.078 (7)	0.072 (6)	0.074 (6)	−0.002 (5)	−0.002 (5)	−0.003 (5)
C11	0.078 (7)	0.082 (7)	0.085 (7)	−0.013 (6)	−0.007 (6)	−0.007 (5)
C12	0.061 (6)	0.097 (7)	0.082 (7)	−0.010 (5)	−0.010 (5)	0.022 (6)
C13	0.056 (6)	0.085 (6)	0.085 (7)	0.011 (5)	0.005 (5)	0.013 (5)
C14	0.057 (6)	0.076 (5)	0.055 (5)	−0.009 (4)	0.012 (4)	0.000 (4)
C15	0.137 (10)	0.059 (5)	0.084 (7)	0.012 (6)	0.036 (7)	0.005 (5)
C16	0.085 (7)	0.055 (5)	0.096 (7)	0.023 (5)	0.016 (6)	0.020 (5)

### Geometric parameters (Å, °)

**Table:**

Cd1—O1	2.230 (5)	C5—C6	1.380 (10)
Cd1—O1^i^	2.230 (5)	C6—C7	1.384 (11)
Cd1—O4^i^	2.295 (5)	C6—H6	0.9300
Cd1—O4	2.295 (5)	C7—H7	0.9300
Cd1—O3^i^	2.315 (5)	C8—C9	1.472 (10)
Cd1—O3	2.315 (5)	C8—H8	0.9300
N1—C8	1.270 (9)	C9—C10	1.377 (11)
N1—C5	1.425 (9)	C9—C14	1.412 (11)
O1—C1	1.248 (8)	C10—C11	1.382 (12)
O2—C1	1.255 (8)	C10—H10	0.9300
O3—C15	1.403 (8)	C11—C12	1.356 (13)
O3—H3	0.8507	C11—H11	0.9300
O4—C16	1.398 (7)	C12—C13	1.401 (13)
O4—H4	0.8668	C12—H12	0.9300
O5—C14	1.352 (10)	C13—C14	1.373 (12)
O5—H5	0.8200	C13—H13	0.9300
C1—C2	1.481 (9)	C15—H15A	0.9600
C2—C7	1.391 (10)	C15—H15B	0.9600
C2—C3	1.400 (9)	C15—H15C	0.9600
C3—C4	1.395 (10)	C16—H16A	0.9600
C3—H3A	0.9300	C16—H16B	0.9599
C4—C5	1.405 (10)	C16—H16C	0.9601
C4—H4A	0.9300		
			
O1—Cd1—O1^i^	180.000 (1)	C5—C6—C7	120.7 (7)
O1—Cd1—O4^i^	90.19 (17)	C5—C6—H6	119.6
O1^i^—Cd1—O4^i^	89.81 (17)	C7—C6—H6	119.6
O1—Cd1—O4	89.81 (17)	C6—C7—C2	121.7 (7)
O1^i^—Cd1—O4	90.19 (17)	C6—C7—H7	119.1
O4^i^—Cd1—O4	180.000 (1)	C2—C7—H7	119.1
O1—Cd1—O3^i^	90.3 (2)	N1—C8—C9	121.4 (8)
O1^i^—Cd1—O3^i^	89.7 (2)	N1—C8—H8	119.3
O4^i^—Cd1—O3^i^	87.23 (19)	C9—C8—H8	119.3
O4—Cd1—O3^i^	92.77 (19)	C10—C9—C14	118.4 (8)
O1—Cd1—O3	89.7 (2)	C10—C9—C8	119.1 (8)
O1^i^—Cd1—O3	90.3 (2)	C14—C9—C8	122.5 (8)
O4^i^—Cd1—O3	92.77 (19)	C9—C10—C11	121.3 (9)
O4—Cd1—O3	87.23 (19)	C9—C10—H10	119.4
O3^i^—Cd1—O3	180.000 (1)	C11—C10—H10	119.4
C8—N1—C5	121.8 (7)	C12—C11—C10	118.9 (9)
C1—O1—Cd1	128.8 (5)	C12—C11—H11	120.5
C15—O3—Cd1	122.5 (4)	C10—C11—H11	120.5
C15—O3—H3	109.5	C11—C12—C13	122.4 (9)
Cd1—O3—H3	115.5	C11—C12—H12	118.8
C16—O4—Cd1	128.8 (4)	C13—C12—H12	118.8
C16—O4—H4	110.1	C14—C13—C12	117.7 (9)
Cd1—O4—H4	107.7	C14—C13—H13	121.1
C14—O5—H5	109.5	C12—C13—H13	121.1
O1—C1—O2	124.0 (7)	O5—C14—C13	118.4 (9)
O1—C1—C2	117.3 (7)	O5—C14—C9	120.5 (7)
O2—C1—C2	118.7 (6)	C13—C14—C9	121.0 (8)
C7—C2—C3	117.3 (7)	O3—C15—H15A	109.5
C7—C2—C1	120.3 (6)	O3—C15—H15B	109.5
C3—C2—C1	122.3 (7)	H15A—C15—H15B	109.5
C4—C3—C2	121.6 (7)	O3—C15—H15C	109.5
C4—C3—H3A	119.2	H15A—C15—H15C	109.5
C2—C3—H3A	119.2	H15B—C15—H15C	109.5
C3—C4—C5	119.4 (7)	O4—C16—H16A	110.1
C3—C4—H4A	120.3	O4—C16—H16B	109.4
C5—C4—H4A	120.3	H16A—C16—H16B	109.5
C6—C5—C4	119.1 (7)	O4—C16—H16C	108.9
C6—C5—N1	116.9 (7)	H16A—C16—H16C	109.5
C4—C5—N1	124.0 (7)	H16B—C16—H16C	109.5
			
O4^i^—Cd1—O1—C1	−171.4 (7)	C3—C4—C5—N1	−178.4 (7)
O4—Cd1—O1—C1	8.6 (7)	C8—N1—C5—C6	157.7 (8)
O3^i^—Cd1—O1—C1	−84.2 (7)	C8—N1—C5—C4	−24.4 (12)
O3—Cd1—O1—C1	95.8 (7)	C4—C5—C6—C7	0.2 (12)
O1—Cd1—O3—C15	−47.5 (5)	N1—C5—C6—C7	178.2 (7)
O1^i^—Cd1—O3—C15	132.5 (5)	C5—C6—C7—C2	0.8 (13)
O4^i^—Cd1—O3—C15	−137.7 (5)	C3—C2—C7—C6	−1.3 (12)
O4—Cd1—O3—C15	42.3 (5)	C1—C2—C7—C6	−179.8 (7)
O1—Cd1—O4—C16	−140.8 (6)	C5—N1—C8—C9	174.8 (7)
O1^i^—Cd1—O4—C16	39.2 (6)	N1—C8—C9—C10	−175.5 (8)
O3^i^—Cd1—O4—C16	−50.5 (6)	N1—C8—C9—C14	3.0 (13)
O3—Cd1—O4—C16	129.5 (6)	C14—C9—C10—C11	−2.9 (13)
Cd1—O1—C1—O2	−2.5 (12)	C8—C9—C10—C11	175.6 (8)
Cd1—O1—C1—C2	178.8 (5)	C9—C10—C11—C12	4.0 (15)
O1—C1—C2—C7	6.8 (11)	C10—C11—C12—C13	−4.0 (16)
O2—C1—C2—C7	−171.9 (8)	C11—C12—C13—C14	3.0 (15)
O1—C1—C2—C3	−171.6 (7)	C12—C13—C14—O5	178.3 (8)
O2—C1—C2—C3	9.7 (11)	C12—C13—C14—C9	−1.9 (14)
C7—C2—C3—C4	1.0 (12)	C10—C9—C14—O5	−178.3 (8)
C1—C2—C3—C4	179.4 (7)	C8—C9—C14—O5	3.2 (13)
C2—C3—C4—C5	−0.1 (13)	C10—C9—C14—C13	1.9 (13)
C3—C4—C5—C6	−0.5 (12)	C8—C9—C14—C13	−176.6 (8)

Symmetry codes: (i) −*x*+2, −*y*+1, −*z*+3.

### Hydrogen-bond geometry (Å, °)

**Table:**

*D*—H···*A*	*D*—H	H···*A*	*D*···*A*	*D*—H···*A*
O4—H4···O2	0.87	1.87	2.653 (7)	150
O5—H5···N1	0.82	1.90	2.632 (9)	148
O3—H3···O2^ii^	0.85	1.83	2.640 (7)	160

Symmetry codes: (ii) −*x*+2, *y*+1/2, −*z*+5/2.
